# The postgraduate medical educational climate assessed by the Danish Residency Educational Climate Test (DK-RECT): a validation and cross-sectional observational study

**DOI:** 10.1186/s12909-023-04909-7

**Published:** 2023-12-12

**Authors:** Rikke Borre Jacobsen, Klarke Boor, Karl Bang Christensen, Vilde Hansteen Ung, Jørn Carlsen, Ole Kirk, Morten Hanefeld Dziegiel, Elsebet Østergaard, Per Rochat, Elisabeth Albrecht-Beste, Marjoes Droogh, Therese S. Lapperre, Fedde Scheele, Jette Led Sørensen

**Affiliations:** 1 Department of Clinical Medicine, Faculty of Health and Medical Sciences, University of Copenhagen, Copenhagen, Denmark; 2 Leiden University Medical Center, Leiden, the Netherlands; 3 Section of Biostatistics, Department of Public Health, University of Copenhagen, Copenhagen, Denmark; 4 Department of Obstetrics and Gynaecology, Wilhelmina Hospital Assen, Assen, the Netherlands; 5 Laboratory of Experimental Medicine and Pediatrics, and Department of Respiratory Medicine, University of Antwerp, Antwerp, Belgium; 6 Amsterdam UMC, Location Vrije Universiteit Amsterdam, Centre for Educational Training, Assessment and Research, Amsterdam, the Netherlands; 7 Department of Anaesthesiology, Operation and Recovery, Trauma Center, and Acute Care, Copenhagen University Hospital, Copenhagen, Rigshospitalet, Entrance 6, 6011 Inge Lehmanns Vej 6, 2100 Copenhagen Ø, Denmark; 8 Juliane Marie Centre and Mary Elizabeth´s Hospital, Copenhagen University Hospital, Copenhagen, Rigshospitalet, Juliane Maries vej 8, 2100 Copenhagen Ø, Danmark

**Keywords:** Education, Medical, Graduate, Factor analysis, Statistical, Psychometrics, Clinical competence, Models, Educational

## Abstract

**Background:**

A good educational climate is essential for delivering high-quality training for medical trainees, professional development, and patient care. The aim of this study was to (1) validate the Dutch Residency Educational Climate Test (D-RECT) in a Danish setting and (2) describe and evaluate the educational climate among medical trainees.

**Methods:**

D-RECT was adopted in a three-step process: translation of D-RECT into Danish (DK-RECT), psychometric validation, and evaluation of educational climate. Trainees from 31 medical specialties at Copenhagen University Hospital – Rigshospitalet, Denmark were asked to complete an online survey in a cross-sectional study.

**Results:**

We performed a forward-backward translation from Dutch to Danish. Confirmatory factor analysis showed that DK-RECT was robust and valid. The reliability analysis showed that only seven trainees from one specialty were needed for a reliable result. With 304 trainees completing DK-RECT, the response rate was 68%. The subsequent analysis indicated a positive overall educational climate, with a median score of 4.0 (interquartile range (IQR): 3.0–5.0) on a five-point Likert scale. Analysis of the subscales showed that the subscale Feedback received the lowest ratings, while Supervision and Peer collaboration were evaluated highest.

**Conclusions:**

Psychometric validation of D-RECT in a Danish context demonstrated valid results on the educational climate in specialist training. DK-RECT can be used to evaluate the effectiveness of interventions in the future and can facilitate the conversation on the educational climate.

**Supplementary Information:**

The online version contains supplementary material available at 10.1186/s12909-023-04909-7.

## Take-home message

**Table:** 

• The Dutch Residency Educational Climate Test (D-RECT), psychometric validated in a Danish context, resulted in a valid and reliable Danish Residency Educational Climate Test (DK-RECT).
• Analysis showed that 2–7 trainees are required to provide reliable educational climate data.
• Analysis of the 11 subscales showed that Feedback had the lowest score, while Supervision and Peer collaboration had the highest.
• DK-RECT, by measuring evaluation of the educational climate, can support quality improvement initiatives by evaluating the effectiveness of interventions in the future and can facilitate the conversation on the educational climate.

## Background

Educational climate in hospitals and postgraduate programmes is essential for delivering high-quality training, professional development, and patient care [1].

In this paper, the term educational climate refer to “trainee perceptions of the formal and informal aspects of education” [2], including the prevailing tone in the clinical educational environment [3]. The educational climate can broadly indicate how well clinical postgraduate education function [4]. The educational context, e.g., organization, setting, coaching, assessment, peer collaboration, practices, and procedures, is also of importance. There is ample evidence that a supportive educational climate in medical postgraduate education is beneficial to professional development. The educational climate is intertwined with the curriculum and if the climate is not supportive, it will be difficult for trainees to successfully go through the training [3, 5–7]. In Denmark, postgraduate programmes in all specialties are based on a well-structured, competence-based curriculum.

Workplace-based training is fundamental in postgraduate specialist training [4], since learning to become a medical specialist involves working and acting as a specialist [8]. In addition to a string focus on patient care, there should also be attention to education, and it is the key to make sure, that education is not overshadowed by patient case duties [9].

Research shows that the educational climate affects learner motivation and self-confidence, influencing outcomes such as academic achievement [10]. A positive educational climate supports the optimal application of knowledge, effective learning, and prevention of stress and burnout [11, 12]. Moreover, improving the quality of the educational climate may lead to improved quality of life and as well as professional performance in trainees [11, 13].

It is challenging to describe and evaluate educational climate [4], and it is difficult to distinguish from culture, with the two terms often used interchangeably. Glisson, a leading researcher in the field, differentiates between organizational culture and organizational climate, referring to the former as the shared behavioural expectations and norms in a work environment and the collective view of the way work is done, while the latter represents staff perceptions of the impact of the work environment on the individual, how it feels to work at the department, e.g. whether it is supportive or stressful [14]. This paper deliberately focus on educational climate, which we define in alignment with Glisson´s organizational climate, as trainees´ perceptions of their educational climate, The instrument we chose has to measure their trainees´ daily experiences and reflections on a continuum from training to daily work, including choices made regarding personal educational needs. Thus, based on Glisson´s definition, examining organizational culture is beyond the scope of this paper.

**Fig. 1:**
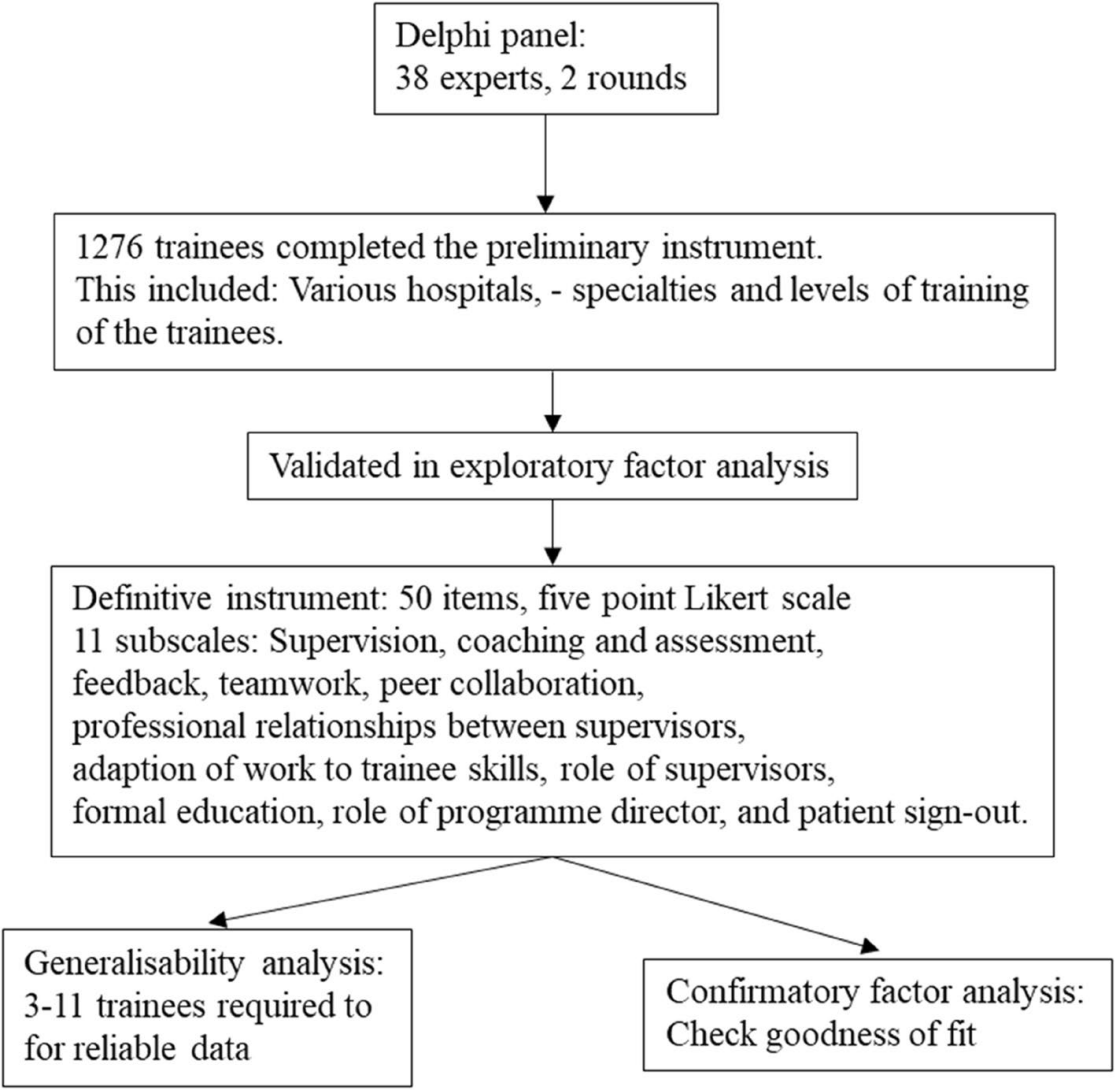
Development of the Dutch Residency Educational Climate Test in 2009. Modified from Boor et al. 2011. Labels of each items and subscales are visualised in Table 3

In order to apply a psychometrically solid and valid evaluation method, we chose the Dutch Residency Educational Climate Test (D-RECT), which was developed de novo primarily to evaluate the postgraduate educational climate, using various methodological approaches, e.g. qualitative research, a Delphi panel, and questionnaires (Figure 1). D-RECT is also based on research from a variety of specialties with different levels of specialized patient care [9] as is the case at Copenhagen University Hospital, Rigshospitalet, where the present study was conducted. Several other instruments [9] also evaluate educational climate for example the Postgraduate Hospital Education Environment Measure PHEEM, but its theoretical foundation is not clearly described and its underlying factor structure is disputed [15].

To date, D-RECT has been used to evaluate the educational climate in studies in the Netherlands [11, 16], Ireland [17], Germany [18], Colombia [19], the Philippines [20], Saudi Arabia [21], Morocco [22], and Iran [23], as well as by gynaecologic oncologists in Europe [24].

Validated in the Dutch setting [25], D-RECT has been used extensively for evaluation and research purposes [26–28]. Several adjustments to the original structure have been published [26], but both versions are capable of measuring the educational climate, which is why we chose to use the original 50-item D-RECT questionnaire.

For our project, our main objective was to examine the educational climate at Copenhagen University Hospital, Rigshospitalet, Denmark which has never been done systematically before, since no suitable instrument was previously available. D-RECT was developed and validated in the Netherlands [4]. To use the 50-item D-RECT instrument in Danish setting, it was necessary to translate and validate it in the Danish context.

## Methods

### Aim

The aim of this study was to validate the 50-item D-RECT in a Danish setting and describe and evaluate the educational climate among postgraduate medical trainees at Rigshospitalet, a tertiary hospital.

Adopting D-RECT involved a three-step process: 1) translation of D-RECT into Danish; 2) psychometric validation; and 3) evaluation of educational climate using the Danish Residency Educational Climate Test (DK-RECT).

. We added questions on demographics as these information was necessary for the further analysis; the sex and age of the trainee, when the trainee graduated from medical school (from Danish or a foreign country), the specialty, the length of employment in the department and the educational level in postgraduate training.

### Setting and inclusion criteria

Trainees from 31 of Rigshospitalet’s 33 specialties were included and completed the questionnaire, while trainees from psychiatric and forensic medicine were excluded due to differences in educational structure. All participating trainees were in clinical rotations at Rigshospitalet during their postgraduate training programme (Table 1).

**Table 1:** Medical education and training in Denmark

• Six-year pregraduate university education (medical degree)
• One-year mandatory house officer training
• Postgraduate training (39 specialties):
○Introductory specialist training programme (first year)
○ Additional specialist training programmes (2–5 years) comprising rotations in university and regional hospitals
• Few trainees not in specific training position

To avoid small sample sizes that might compromise anonymity and make the statistical analyses inconclusive, trainees were divided into four groups: surgery, medicine, anaesthesiology, and auxiliary. This grouping was done in accordance with the standard curriculum in the specialties and the hospital´s educational organization. According to the East Denmark Regional Board of Postgraduate Medical Education, Rigshospitalet continually has about 400 trainees. In September 2019 the Board invited trainees, identified via an ongoing formal evaluation and quality assurance programme in postgraduate medical training, to complete DK-RECT online. After receiving two e-mail reminders, non-responders were excluded.

### Statistics – testing and validation

#### Descriptive statistics

Descriptive statistics were used for each DK-RECT item, including range of inter-item correlations and correlation with total subscale score and score of other items in the subscale. Inter-item correlation should be neither too low nor too high to indicate whether an item is representative of not only the subscale but also captures something unique, i.e., that items do not duplicate content in other subscales. Item-total correlation examines whether items correlate well with the total score. High item-total correlation is acceptable.

Trainees, divided into the four aforementioned groups (surgery, medicine, anaesthesiology, and auxiliary) were compared with Kruskal-Wallis test, where P<0.05 indicates a statistical difference between the groups overall.

#### Confirmatory factor analysis (CFA)

CFA, which tests the fit between observed responses and the proposed structure, was used to test whether the items fit the subscale structure. To analyze the five-point Likert scale items we used CFA for ordinal items based on polychoric correlations. This methodology required rescoring of items (collapsing categories 1-3 and 4-5 on the Likert scale), whenever a response category was not observed in a subgroup. We further tested the multidimensional model CFA by studying invariance across sex and the values of a categorised version of the variable length of employment.

We were unable to test the fit across different specialties or the four main groups because some groups were too small and some categories were missing, which made it impossible to collapse them. We then merged the four main groups into two subgroups: medicine - auxiliary and surgery - anaesthesiology. The Supplementary tables and materials provide additional information about the CFA model and analysis.

#### Reliability analysis (internal consistency)

Generalisability theory was used to address validation and reliability, allowing estimation of the size of relevant influences affecting the measurement [29]. We performed a reliability analysis for the mean total score and for each separate subscale to estimate the number of trainees or specialties needed for reliable scores on the department levels. We treated the total number of items as fixed similar to Boor et al., the number of trainees within a single department and the number of departments were allowed to vary [4]. We applied a standard error of measurement (SEM) for a single specialty of <0.26 (1.96 x 0.26 x 2=1.0). We assumed a maximum noise level of 1.0 on a five-point Likert scale and as the smallest admissible value for a 95% confidence interval.

The CFA was conducted using the R package lavaan [30], while SAS 9.4 was used for other statistical analyses.

## Results

### Translation into Danish

Two medical consultants bilingual in Danish and Dutch, who also had in-depth knowledge of trainee programmes and educational traditions in both countries, translated the 50-item D-RECT [31]. The model for forward-backward translation was implemented in a modified and simplified approach, as described by Eremenco et al., because only four persons were responsible for the translation process. The two Danish–Dutch consultants were well educated, trained, and possessed the relevant knowledge about the postgraduate curriculum in both countries and the educational organization in Denmark. One forward translated from Dutch to Danish, while the other back translated the result from Danish to Dutch. After comparing the two versions, the Danish educational terminology was adjusted to identify misleading phrasing. Five trainees subsequently took a pilot test.

### Description of participants

*Population* (Table 2): Questionnaires were manually sorted. Of the 445 trainees contacted, 378 completed DK-RECT, and 74 tests were excluded because two thirds or more of the questions (items) were unanswered. The second column (N) in Table 3 lists the number of items answered. Overall, 304 tests were suitable for analysis (68% response rate), with all 31 specialties represented.

**Table 2:** Baseline characteristics of participants

**Characteristics**	**N**	**%**
Number contacted	445	
Declined participation	67	15
Excluded^a^	74	17
Included	304	68
Sex (female/male)	185/119	61/39
Surgery group^b^	85	28
Medicine group^c^	127	42
Auxiliary group^d^	61	20
Anaesthesiology group^e^	31	10
Medical school (Danish/not Danish)	286/18	94/6
Trainee, year 1	56	18
Trainee, year 2–6	240	79
Trainees not in specific position	8	3

^a^2/3 items left blank

^b^Surgery: obstetrics and gynaecology, vascular and thoracic surgery, urology, neurosurgery, plastic surgery, ophthalmology, otolaryngology, orthopaedic/spinal surgery

^c^Medicine: paediatrics, cardiology, haematology, gastroenterology, endocrinology, neurology, neurophysiology, oncology, rheumatology, infectious diseases, nephrology, pulmonary medicine

^d^Auxiliary: immunology, clinical physiology, nuclear medicine, pathology, clinical chemistry, clinical microbiology, radiology, clinical genetics

^e^Anaesthesiology: Including intensive care

**Table 3:** Validation of 50-item DK-RECT^a^ with 11 subscales and evaluation of postgraduate educational climate

**Subcales; No. 1-11**		**N**	**Median (IQR)**^**b**^	**Mean(SD)**	**Floor/ Ceiling (%)**^**c**^	**Range inter-item correlation**	**Item-total correlation**^**d**^	**Item-restcore correlation**^**e**^
**Items; No. 1-50**								
**1. Supervision**								
1	I can always get a hold of a supervisor if I need to	304	5.0 (4.0-5.0)	4.3(0.9)	1/52.3	(0.6-0.8)	0.9	0.7
2	A supervisor is easily accessible if I have the need to discuss something	304	5.0 (4.0-5.0)	4.2(1.0)	1/50.3	(0.6-0.8)	0.9	0.8
3	There is a clear understanding of when I should ask for guidance	303	4.0 (4.0-5.0)	3.6(1.1)	2.3/27.1	(0.5-0.6)	0.8	0.6
**2. Coaching and assessment**								
4	I am regularly asked to provide a rationale for my patient care and treatment	283	3.0 (2.0-4.0)	3.3(1.1)	5/15.6	(0.4-0.6)	0.7	0.6
5	I receive guidance on communicating with demanding patients	274	3.0 (2.0-4.0)	3.1(1.0)	5.9/5.1	(0.4-0.5)	0.7	0.6
6	My supervisors take the initiative to explain their patient care and treatment	281	3.0 (2.0-4.0)	3.3(1.1)	7.9/12.5	(0.4-0.6)	0.7	0.6
7	My supervisors, unasked, tell me how I am performing	302	3.0 (2.0-4.0)	3.1(1.1)	9.3/11.6	(0.4-0.7)	0.7	0.7
8	Supervisors take the initiative to discuss difficult situations I have been involved in	290	3.0 (2.0-4.0)	3.1(1.1)	7.2/11.7	(0.4-0.7)	0.7	0.6
9	Supervisors assess whether the patient care I perform corresponds to my level of training	292	4.0 (3.0-4.0)	3.7(1.0)	4.5/19.5	(0.4-0.7)	0.7	0.6
10	Supervisors occasionally observe when patient medical histories are taken	249	2.0 (1.0-3.0)	2.5(1.2)	25.7/6.0	(0.4-0.5)	0.6	0.5
11	Supervisors assess not only my medical expertise but also other skills, such as collaborative, organisational, or professional abilities	299	4.0 (3.0-4.0)	3.5(1.0)	5.4/16.1	(0.4-0.6)	0.6	0.5
**3. Feedback**								
12	Supervisors give me feedback on both what I do right and what I can improve	302	4.0 (3.0-4.0)	3.6(1.1)	5.0/15.9	(0.5-0.5)	0.7	0.4
13	Structured forms are used to provide feedback	293	3.0 (2.0-4.0)	2.7(1.2)	18.4/7.9	(0.5-0.9)	0.9	0.8
14	Structured observation forms are used to clarify my progress	294	3.0 (2.0-4.0)	2.7(1.1)	17.7/6.5	(0.5-0.9)	0.9	0.8
**4. Teamwork**								
15	Specialist doctors, nurses, other health professionals and trainees work together in teams in my department	297	5.0 (4.0-5.0)	4.3(0.8)	0.3/51.9	(0.4-0.5)	0.8	0.6
16	Nurses and other health professionals contribute positively to my training	303	4.0 (4.0-5.0)	4.1(0.8)	0.3/34	(0.4-0.7)	0.8	0.6
17	Nurses and other health professionals are willing to reflect jointly on patient care	289	4.0 (4.0-5.0)	4.2(0.8)	0.0/39.5	(0.4-0.7)	0.8	0.6
18	We explicitly discuss our teamwork in my training	298	3.0 (2.0-4.0)	3.1(1.0)	5.0/8.7	(0.37-0.43)	0.7	0.4
**5. Peer collaboration**								
19	There is high-quality collaboration between the trainees in my department	301	5.0 (4.0-5.0)	4.5(0.7)	0.7/56.5	(0.5-0.7)	0.8	0.5
20	As trainees we jointly ensure that the day’s work is completed	301	4.0 (4.0-5.0)	4.2(0.9)	1.0/45.2	(0.5-0.7)	0.8	0.6
21	Junior doctors seamlessly switch and cover calls among themselves when necessary	292	4.0 (4.0-5.0)	4.1(0.8)	1.0/36.3	(0.48-0.49)	0.8	0.5
**6. Professional relationship between supervisors**								
22	Continuity of care is unaffected by conflicts between supervisors	255	4.0 (4.0-5.0)	4.0(0.9)	0.4/34.9	(0.6-0.7)	0.7	0.5
23	Differences of opinion between supervisors about patient management are discussed instructively in the team	258	4.0 (3.0-4.0)	3.9(0.9)	1.6/24.8	(0.6-0.7)	0.7	0.5
24	Serious conflicts in the department do not negatively affect the working environment	286	4.0 (3.0-5.0)	3.8(1.1)	4.9/26.8	(0.58-0.62)	0.7	0.5
**7. Work adapted to trainee skill level**								
25	The work that I do corresponds to my level of experience	304	4.0 (4.0-5.0)	4.0(0.9)	0.3/29.6	(0.3-0.6)	0.7	0.5
26	The work that I do corresponds to my current learning objectives in my training	304	4.0 (3.0-5.0)	3.9(0.9)	0.7/27.6	(0.4-0.6)	0.8	0.6
27	I get the opportunity to follow-up on patients	284	4.0 (3.0-5.0)	3.8(1.1)	3.2/27.1	(0.3-0.5)	0.7	0.5
28	There is enough time in the schedule for me to learn new skills	297	3.0 (2.0-4.0)	3.4(1.2)	5.7/20.9	(0.4-0.5)	0.8	0.5
**8. Role of supervisors**								
29	My supervisors take the time to explain when asked for advice	302	4.0 (4.0-5.0)	4.3(0.8)	1/43.1	(0.5-0.7)	0.8	0.7
30	My supervisors are willing to discuss patient care and treatment	292	4.0 (4.0-5.0)	4.3(0.9)	1.0/45.9	(0.5-0.7)	0.8	0.7
31	There are no supervisors who have a negative impact on the educational environment	297	4.0 (3.0-5.0)	3.7(1.1)	4.0/26.4	(0.4-0.7)	0.8	0.7
32	My supervisors are interested in me as a person	302	4.0 (3.0-5.0)	3.8(1.0)	1.6/27.3	(0.5-0.7)	0.8	0.7
33	My supervisors treat me with respect	299	4.0 (4.0-5.0)	4.1(0.9)	1.7/36.5	(0.5-0.8)	0.9	0.8
34	My supervisors are all in their own way positive role models	300	4.0 (4.0-5.0)	4.0(1.0)	2.0/33.1	(0.5-0.8)	0.8	0.8
35	The amount of supervision is adapted to my level of experience	302	4.0 (3.0-5.0)	3.8(1.0)	0.0/27.3	(0.5-0.7)	0.8	0.7
36	It is clear to me who supervises my work	302	4.0 (3.0-5.0)	3.8(1.2)	3.3/32.9	(0.4-0.7)	0.7	0.6
**9. Formal education**								
37	Trainees are normally able to attend scheduled educational activities	300	4.0 (4.0-5.0)	4.0(1.0)	2.0/33.4	(0.6-0.7)	0.8	0.7
38	Scheduled educational activities are carried out	300	4.0 (4.0-5.0)	4.1(0.9)	1.3/35.1	(0.68-0.72)	0.9	0.8
39	Supervisors actively contribute to developing and planning high-quality teaching	301	4.0 (3.0-5.0)	3.8(1.1)	3.0/26.7	(0.6-0.8)	0.9	0.8
40	Formal teaching at the department is well-suited to my needs	300	4.0 (4.0-5.0)	3.9(1.0)	1.3/27.2	(0.6-0.8)	0.9	0.8
**10. Role of programme director**								
41	My programme director knows how far I am in my formal training	298	4.0 (4.0-5.0)	4.3(0.9)	0.7/46.9	(0.5-0.8)	0.7	0.6
42	My programme director provides other supervisors with guidance when needed	273	4.0 (3.0-5.0)	3.6(1.1)	1.1/25.9	(0.5-0.7)	0.8	0.7
43	My programme director works actively to achieve high-quality education	298	4.0 (4.0-5.0)	4.2(1.1)	1.7/46.2	(0.6-0.8)	0.7	0.6
44	We constructively discuss my performance during the departmental assessment interview	276	4.0 (3.0-5.0)	3.9(1.1)	3.2/30.6	(0.6-0.8)	0.8	0.7
45	My plans for the future are reviewed during the assessment interview	282	4.0 (3.0-5.0)	3.9(1.1)	1.4/29.3	(0.5-0.8)	0.8	0.7
46	Assessments of my work by various supervisors are considered during the assessment interview	271	4.0 (3.0-4.0)	3.4(1.2)	5.8/21.4	(0.5-0.7)	0.8	0.7
**11. Patient sign-out**								
47	If the treatment plan I developed with my supervisor is criticised after a morning briefing, I am confident that my supervisor will back me up	250	4.0 (4.0-5.0)	4.0(1.0)	1.2/27.8	(0.6-0.7)	0.8	0.7
48	There is a safe climate at morning briefings	294	4.0 (4.0-5.0)	4.1(1.0)	1.8/36.8	(0.66-0.73)	0.8	0.7
49	Morning briefings are also used as a teaching opportunity	293	4.0 (4.0-5.0)	4.1(1.0)	1.3/38.9	(0.6-0.7)	0.8	0.6
50	Supervisors encourage trainees to join in discussions at morning briefings	291	4.0 (3.0-5.0)	3.7(1.1)	2.0/27.0	(0.6-0.7)	0,8	0.6

^a^Based on Dutch Residency Educational Climate Test; rated on five-point Likert scale and translated into English for this paper. Likert scale (answer categories: 1=totally disagree, 2=disagree, 3= neutral, 4=agree, 5=totally agree)

^N^Respondents per item

^b^Median for individual items

^c^Percentage selecting lowest and highest reliable Likert rating

^d^Examines whether items correlate well with total score; high correlation indicates items measure same latent variable

^e^Examines how much the score of one subscale item relates to other item scores in same subscale and measure subscale consistency and reliability

The response rate per item was at least 82%. During data collection most trainees (79%) had completed half of their postgraduate training and had worked at Rigshospitalet for an average of 13.1 months (SD: 10.3 months).

### Development and validation

Generally, the item-total correlation was high (Table 3), with low correlation (=0.6) for only two items (10 and 11) in the Coaching and assessment subscale. Overall, our multidimensional CFA model showed that participant answers were reliable and independent of sex and length of employment (Supplementary tables and materials). CFA of the 50-item DK-RECT fit (outcome of the Close Fit Index and Trucker-Lewis Index was ≥0.95) and confirmed validity of D-RECT’s factor structure [4].

Our generalisability test showed that for one department, SEM based on the overall score for the 11 subscales, required that 2-7 trainees responded to the questionnaire to achieve a reliable inference of one point (Supplementary tables and materials). In one department, seven trainees were needed for reliable outcomes for every subscale. For groups of specialties, eight different specialties, each with two trainees, were required for a reliable total score (Table 4).

**Table 4:** Reliability analysis of Danish Residency Educational Climate Test of number of trainees and specialties required for reliable results^a^

**Subscales**	**SEM**^b^** (one department)** **n (trainees)**	**RMSE**^c^** (group of specialties)** **n (specialties)/ n (trainees)**
1. Supervision	6	5/2
2. Coaching and assessment	3	No^d^
3. Feedback	7	8/2
4. Teamwork	4	4/2
5. Peer collaboration	4	3/2
6. Professional relationship between supervisors	5	7/2
7. Work adapted to trainee skill level	4	4/2
8. Role of supervisors	2	No^d^
9. Formal education	3	No^d^
10. Role of programme director	3	No^d^
11. Patient sign-out	3	No^d^

^a^E.g. in the supervision subscale, including six trainees from one specialty or two trainees each from five specialties achieved more reliable results

^b^SEM: Standard error of measurement

^c^RMSE: Root mean square error

^d^Additional specialties added no benefit

### DK-RECT results

The mean DK-RECT score was 3.8 (SD 1.1) (median 4(IQR): 3.0-5.0), while the median individual item score was **≥**3.0 (Table 3), except for item 10 (supervisors occasionally observe when patient medical histories are taken), which was 2.0 (IQR: 1.0-3.0). Table 5 shows the overall rating for each subscale for the specialty groups. The educational climate for the 11 subscales was acceptable (median score **≥**3.0), with only subscale 3 (Feedback) rated lower (median 3.0 (IQR: 2.3-3.8). The non-parametric tests showed significant differences across the four specialty groups for: Feedback, Coaching and assessment, Teamwork, Professional relationship between supervisors, and Work adapted to trainee skill level.

**Table 5:** Evaluation of educational climate among the four specialty groups

**Subscales**	**Median (IQR)**^a^	**Mean (SD)**	**P**^**2**^
**1. Supervision**	4.3(3.7-4.7)	4.1(1.0)	*P*=0.4147
Surgery	4.3(3.7-4.7)	4.0(1.1)	
Medicine	4.3(3.3-4.7)	4.0(1.1)	
Auxiliary	4.3(3.7-4.7)	4.1(0.9)	
Anaesthesiology	4.7(4.0-5.0)	4.3(1.0)	
**2. Coaching and assessment**	3.3(2.6-3.9)	3.2(1.2)	*P*=0.0065
Surgery	3.1(2.5-3.8)	3.1(1.2)	
Medicine	3.1(2.6-3.8)	3.1(1.1)	
Auxiliary	3.6(2.9-4.0)	3.4(1.0)	
Anaesthesiology	3.7(2.9-4.0)	3.6(1.1)	
**3. Feedback**	3.0(2.3-3.8)	3.0(1.2)	*P*<0.0001
Surgery	3.0(2.0-3.7)	2.9(1.2)	
Medicine	2.7(2.0-3.3)	2.7(1.2)	
Auxiliary	3.3(2.7-4.0)	3.3(1.2)	
Anaesthesiology	3.7(3.0-4.0)	3.6(1.1)	
**4. Teamwork**	4.0(3.5-4.5)	3.9(0.7)	P<0.0001
Surgery	4.0(3.8-4.5)	4.0(0.7)	
Medicine	4.0(3.5-4.5)	3.9(0.7)	
Auxiliary	3.7(3.3-4.3)	4.0(0.7)	
Anaesthesiology	4.3(4.0-5.0)	4.4(0.6)	
**5. Peer collaboration**	4.3(4.0-5.0)	4.3(0.7)	*P*=0.0672
Surgery	4.3(4.0-4.7)	4.2(0.7)	
Medicine	4.3(4.0-5.0)	4.4(0.6)	
Auxiliary	4.7(4.0-5.0)	4.4(0.7)	
Anaesthesiology	4.0(3.5-4.7)	4.0(0.9)	
**6. Professional relationship between supervisors**	4.0(3.3-4.5)	3.8(0.9)	*P*=0.0001
Surgery	4.0(3.7-4.5)	4.0(0.8)	
Medicine	4.0(3.3-4.7)	3.9(0.8)	
Auxiliary	3.3(2.7-4.0)	3.3(1.1)	
Anaesthesiology	4.0(4.0-5.0)	4.1(0.8)	
**7. Work adapted to trainee skill level**	3.8(3.3-4.3)	3.8(0.8)	*P*=0.0225
Surgery	3.8(3.0-4.3)	3.7(0.8)	
Medicine	3.8(3.0-4.3)	3.7(0.8)	
Auxiliary	4.0(3.3-4.3)	3.9(0.7)	
Anaesthesiology	4.0(3.8-4.5)	4.1(0.6)	
**8. Role of supervisors**	4.0(3.5-4.6)	4.0(0.7)	*P*=0.3307
Surgery	4.1(3.7-4.6)	4.1(0.7)	
Medicine	4.0(3.5-4.5)	3.9(0.7)	
Auxiliary	4.0(3.5-4.6)	3.9(0.8)	
Anaesthesiology	4.0(3.8-4.6)	4.1(0.6)	
**9. Formal education**	4.0(3.5-4.5)	4.0(0.8)	*P*=0.2525
Surgery	4.0(3.5-4.4)	3.9(0.8)	
Medicine	4.0(3.8-4.8)	4.1(0.7)	
Auxiliary	4.0(3.3-4.5)	3.8(0.9)	
Anaesthesiology	4.0(3.5-4.8)	4.0(0.9)	
**10. Role of programme director**	4.0(3.5-4.5)	4.0(0.7)	*P*=0.3606
Surgery	4.0(3.7-4.7)	4.1(0.7)	
Medicine	4.0(3.5-4.4)	3.9(0.7)	
Auxiliary	3.8(3.3-4.7)	3.9(0.8)	
Anaesthesiology	4.0(3.5-4.5)	4.0(0.7)	
**11. Patient sign-out**	4.0(3.5-4.5)	4.0(0.7)	*P*=0.7115
Surgery	4.0(3.5-4.5)	4.0(0.7)	
Medicine	4.0(3.8-4.8)	4.1(0.7)	
Auxiliary	4.0(3.5-4.6)	4.0(0.8)	
Anaesthesiology	4.0(3.5-5.0)	4.1(0.8)	

^a^Median of subscale mean scores

^2^Kruskal-Wallis test; P<0.05 indicates statistical difference in four specialty groups overall

## Discussion

### Main findings

This study indicates that the 50-item DK-RECT is a reliable instrument in a Danish tertiary hospital to examine the educational climate of medical trainees. DK-RECT’s internal consistency is high, the psychometric analysis showing robustness and validity. Moreover, only 2-7 trainees were required from each specialty for reliable results. With a high overall mean rating score DK-RECT showed that the educational climate was good but that some specialties had potential for improvement, particularly in Feedback and Coaching and assessment.

### DK-RECT development and validation

CFA of DK-RECT showed that the content of each item was representative and captured unique features. Validation of the DK-RECT questionnaire, as observed by Boor et. al. (2011) for the 50-item D-RECT, and also concluded by Silkens et. al. (2016) for the 35-item D-RECT, was acceptable, despite the fact that the 35-item D-RECT instrument was slightly different and validated in a different context.

There was acceptable homogeneity among individual items and DK-RECT as a whole; no items were unnecessary. The high response rate per item indicates that the trainees used all items, and that the full five-point Likert scale was used. The wording of the questionnaire was important, and although the dual, in-depth translation process was time-consuming, the adaptation turned out to be an essential prerequisite for the analysis and psychometric validation.

Like the Dutch study [4], CFA showed that items did not display differential item functioning (Supplementary tables and materials), indicating that neither the sex of the trainees, the number of years of work, nor whether they belonged to the medicine or surgery group, were predictive of a pattern in how they answered the questions.

Notably, the generalisability analysis (Table 4) showed that 2-7 replies per item in one specialty were sufficient per subscale to achieve a reliable inference of one point and reliable results. This is highly comparable to Boor et al.´s results (2011). Hence, including additional specialties or several trainees from each specialty offers no benefit in achieving more reliable results. This means that specialties with few trainees can assess their educational climate without identification of the responders, confirming the feasibility of DK-RECT. However, ensuring the anonymity of trainees in small departments can be challenging, which is why adding trainees from two or more departments may be necessary. The present study reported specialties in four groups to maintain the full anonymity of respondents.

The 50-item D-RECT instrument, which is now 15 years old, was validated after its introduction in several contexts and performed well, which strengthens the argument for its use. However, it also indicates how important it is to perform validation studies before implementing a tool. One well-known revision was Silkens et al.´s 35-item shorter version [25], which was developed because some items in the original 50-item version performed poorly and seemed outdated. They concluded that the nine-factor model with 35 items fitted better, with an improvement in the Close Fit Index and Trucker-Lewis Index [25]. But even though the exact number of items and the clustering might vary, the 50-items D-RECT is still valid and the results reliable.

Researchers must be aware that the educational climate can change over time and that trainee perceptions and expectations of the ideal educational climate may vary with new and younger trainees. These factors indicate instruments must be re-validated over time and items revised since they may lose relevance, also due to organizational changes or the impact of new developments or initiatives designed to strengthen the educational climate.

### Evaluation of postgraduate educational climate

A comparison of DK-RECT with the Dutch mean scores showed that most trainees evaluated the educational climate positively, which is comparable with the Dutch results: 3.8 (SD 0.3) [4]; 3.5 (SD 0.4) [11]; and 3.9 (SD 0.4) [25], even allowing for the fact that the instrument differed lightly in Silkens et.al 2016 study.

Most DK-RECT subscales were positively evaluated, while those with mean scores <3.9 mainly concerned the organization of the education (Teamwork, Professional relationship between supervisors, and Work adapted to trainee skill level), and with supervisor behaviour (Coaching and assessment, and Feedback). The lowest scores were for Feedback (item 13: Structured forms are used to provide feedback, and item 14: Structured observation forms are used to clarify my progress), with a median score of 3.0 (SD 2.0-4.0) and Coaching and assessment (item 10: Supervisors occasionally observe when patient medical histories are taken), with a median score of 2.0 (SD 1.0-3.0). Especially these two subscales showed significant differences across specialty groups.

The Feedback subscale scored lowest overall, with specialty groups differing significantly (Table 5). This should be a matter of concern, because even though providing feedback is a complex, subtle interaction influenced by multiple factors such as the supervisor, message, delivery method, and supervisor-learner relationship [32], immediate, specific, and frequent feedback is clearly vital to successful trainee progression and professional development. This is why it should optimally take place at department level and be benchmarked against others at the hospital. Thus, detecting outliers in DK-RECT measurements offers opportunities to provide remediation in low performing departments and learn from high performers.

Work adapted to trainee skill level was also rated low, with specialty groups differing significantly (Table 5). Raising awareness about trainee educational programmes is beneficial because a good educational climate positively affects teaching faculty [1]. This awareness can involve aligning trainee and supervisor expectations concerning complex patient cases, increasing their familiarity with learning objectives, and acknowledging how time-consuming learning new skills is.

### Strengths and limitations

It is a strength that the response rate was high (68%), likely because it was web-based, written in respondents’ native language, and with anonymous participation. Also, data collection was user friendly, and the heads of education in each specialty frequently communicated about the response rate.

Non-responder bias nonetheless remains an issue but was not examined further.

Finally, there are some caveats to consider: first, identifying participants who met our criteria was difficult for all specialties, regardless of the time of data collection since rotations affected whether a trainee worked in a specialty for only a few weeks or months. Although the average length of employment was 13.1 months, the SD was broad, indicating that some clinical rotations lasted only a few months, possibly influencing trainee perception of the educational climate compared to those employed for over a year. This issue can be addressed, either by conducting the survey at a time fixed in relation to clinical rotations, or by analysing the results according to the duration of the clinical rotations. Second, the term supervisor can potentially cause confusion in the translated questionnaire. Trainee comments indicated difficulties in answering questions because the term was used to describe both clinical supervisor and the head of education. The next version of DK-RECT must address this issue. Third, specialties with little direct patient contact questioned the relevance of certain items on, e.g patient care, and especially item 10 (Supervisors occasionally observe when patient medical histories are taken), as subsequently reflected in the low item-total correlation and the floor effect (Table 3). This is in contrast to a previous study [25], which argues that psychometric validation means the test can be used in various postgraduate settings in teaching and non-teaching hospitals and with and without patient care-related aspects. Thus, auxiliary specialties need greater attention in future discussions on the revision of residency educational climate tests.

## Conclusions, perspectives, implications, and future research

This study addresses the lack of validated instruments available in Danish with a documented high internal consistency for supporting the development of the educational climate and including formative evaluation. The validated DK-RECT is useful for measuring the educational climate in postgraduate training by offering a standardised, objective method for comparing various educational contexts, e.g., whether differences in subscales and items between specialties are associated with better training and better patient outcomes. DK-RECT allows accurate evaluation of whether curricular and educational changes lead to improvement. We chose to use Boor et al.´s original 50-item D-RECT instrument [4], but Silkens et al.´s 35-item instrument  [25], could also have been applied, though should be validated again if used in future studies in Denmark.

One issue for further exploration is how frequently evaluation of the educational climate should be done. Too frequent evaluation can cause a substantial decline in response rate due to participant response fatigue. Positive results and progress may simply reflect the increased attention paid to the quality of the educational climate [25], i.e., the Hawthorne effect [33]. Even if departments generally continually work to improve the educational climate, curriculum changes may not result in convincing improvements for years. Of note, departments with lower scores may feel pressured to improve the educational climate, whereas higher scores may be a disincentive [25]. The first version of DK-RECT provides the opportunity to establish educational climate benchmarks, allowing comparisons between hospitals in Denmark and abroad.

Developed in 2009, D-RECT did not have access to today’s information and communication technology, social media, digital learning, and simulations. Patient involvement in medical education also represents a valuable way to improve learning. Consequently, updating and further developing D-RECT and DK-RECT is warranted.

### Supplementary Information

**Additional file 1:** **Table S1.** Standard error of measurement (SEM) and number of trainees required to achieve a reliable outcome for one department. **Table S2**. Fit of multidimensional CFA.

## Data Availability

The datasets generated and/or analysed in the current study are not publicly available due to anonymity requirements but are available from the corresponding author upon reasonable request. DK-RECT is also available from the corresponding author by mail upon request.

## Abbreviations

CFA: Confirmatory factor analysis
CFI: Close Fit Index
D-RECT: Dutch Residency Educational Climate Test
DK-RECT: Danish Residency Educational Climate Test
DIF: Differential item functioning
IQR: Interquartile range
SEM: Standard error of measurement
