# Supplementary material for: The postgraduate medical educational climate assessed by the Danish Residency Educational Climate Test (DK-RECT): a validation and cross-sectional observational study
Source: BMC Med Educ. 2023 Dec 12;23:943. doi: 10.1186/s12909-023-04909-7 (PMC10717804; doi:10.1186/s12909-023-04909-7)
Supplement: Supplementary file 1 — Additional file 1: Table S1. Standard error of measurement (SEM) and number of trainees required to achieve a reliable outcome for one department. Table S2. Fit of multidimensional CFA. [file 12909_2023_4909_MOESM1_ESM.docx]

**Supplementary tables and materials to the paper**

## **The postgraduate medical educational climate assessed by the Danish Residency Educational Climate Test (DK-RECT):** **A validation and cross-sectional observational study**

Rikke Borre Jacobsen^1*^, Klarke Boor^2^, Karl Bang Christensen^3^, Vilde Hansteen Ung^3^, Jørn Carlsen^1^, Ole Kirk^1^, Morten Hanefeld Dziegiel^1^, Elsebet Østergaard^1^, Per Rochat^1^, Elisabeth Albrecht-Beste^1^, Marjoes Droogh^1,4^, Therese S. Lapperre^1,5^, Fedde Scheele^6^, Jette Led Sørensen^1^

^1^Department of Clinical Medicine, University of Copenhagen, Copenhagen, Denmark

^2^Leiden University Medical Center, Leiden, The Netherlands

^3^Section of Biostatistics, Department of Public Health, University of Copenhagen, Copenhagen, Denmark

^4^Department of Obstetrics and Gynaecology, Wilhelmina Hospital Assen, Assen, the Netherlands

^5^Laboratory of Experimental Medicine and Pediatrics, and Department of Respiratory Medicine, University of Antwerp, Antwerp, Belgium

^6^Amsterdam UMC, Location Vrije Universiteit Amsterdam, Centre for Educational Training, Assessment and Research, Amsterdam, The Netherlands

## **Reliability analysis of DK-RECT**

| **Table S1.** Standard error of measurement (SEM) and number of trainees required to achieve a reliable outcome for one department. | | | | | | | | | | |  |
| --- | --- | --- | --- | --- | --- | --- | --- | --- | --- | --- | --- |
| **Subscales** | **1*** | **2*** | **3*** | **4*** | **5*** | **6*** | **7*** | **8*** | **9*** | **10*** | |
| 1. Supervision | 0.58 | 0.41 | 0.33 | 0.29 | 0.26 | ***0.24*** | 0.22 | 0.20 | 0.19 | 0.18 | |
| 2. Coaching and assessment | 0.38 | 0.27 | ***0.22*** | 0.19 | 0.17 | 0.16 | 0.14 | 0.13 | 0.13 | 0.12 | |
| 3. Feedback | 0.64 | 0.45 | 0.37 | 0.32 | 0.29 | 0.26 | ***0.24*** | 0.23 | 0.21 | 0.20 | |
| 4. Teamwork | 0.47 | 0.34 | 0.27 | ***0.24*** | 0.21 | 0.19 | 0.18 | 0.17 | 0.16 | 0.15 | |
| 5. Peer collaboration | 0.45 | 0.32 | 0.26 | ***0.22*** | 0.20 | 0.18 | 0.17 | 0.16 | 0.15 | 0.14 | |
| 6. Professional relationship between supervisors | 0.52 | 0.36 | 0.30 | 0.26 | ***0.23*** | 0.21 | 0.20 | 0.18 | 0.17 | 0.16 | |
| 7. Work is adapted to trainee skill level | 0.50 | 0.35 | 0.29 | ***0.25*** | 0.22 | 0.20 | 0.19 | 0.18 | 0.17 | 0.16 | |
| 8. Role of supervisors | 0.31 | ***0.22*** | 0.18 | 0.16 | 0.14 | 0.13 | 0.12 | 0.11 | 0.10 | 0.10 | |
| 9. Formal education | 0.43 | 0.30 | ***0.25*** | 0.21 | 0.19 | 0.17 | 0.16 | 0.15 | 0.14 | 0.14 | |
| 10. Role of programme director | 0.39 | 0.27 | ***0.22*** | 0.19 | 0.17 | 0.16 | 0.15 | 0.14 | 0.13 | 0.12 | |
| 11. Patient sign-out | 0.44 | 0.31 | ***0.25*** | 0.22 | 0.19 | 0.18 | 0.16 | 0.15 | 0.15 | 0.14 | |

^*^Trainee (n)

SEM refers to the standard deviation (SD) of scores for a single trainee if tested multiple times. The 0.26 SEM allowed reliably differentiating one point on the Likert scale.

## **Confirmatory factor analysis and differential item functioning**

R package Lavaan (1) was used in the confirmatory factor analysis (CFA) of ordinal items. Differential item functioning (DIF) (2) was tested using multi-group CFA (3). An item displayed DIF if variables like sex and age systematically affected the responses, e.g., if women systematically scored higher on an item than men with the same overall subscale score.

The CFA analysis reported the chi-squared test of the model fit. If the P-value was <0.05, the proposed model did not fit. The fit of CFA models was also evaluated using the root mean square error of approximation (RMSEA), corresponding 90% confidence interval, the Close Fit Index (CFI) and the Tucker‐Lewis Index (TLI). The following criteria were used: RMSEA ≤0.05 indicated a good data fit, and >0.05 but <0.08 a satisfactory fit (4). RMSEA was most suitable for models with many degrees of freedom (5). Values ≥0.95 for CFI and TLI indicated a good fit.

Table S2, which shows the fit of the multidimensional CFA model, confirms the validity of the factor structure (6). To analyze the five-point Likert scale items we used CFA for ordinal items based on polychoric correlations. This methodology required rescoring of items (collapsing categories 1-3 and 4-5 on the Likert scale), whenever a response category was not observed in a subgroup.We further tested the multidimensional model CFA by studying invariance across sex and the values of a categorised version of the variable length of employment.
We were unable to test the fit across different specialties or the four main groups because some groups were so small that many categories were missing, making it impossible to collapse them. We then merged the four main groups into two subgroups (medicine and auxiliary) and (surgery and anaesthesiology). Table S2 shows no problems with DIF.

| **Table S2.** Fit of multidimensional CFA. | | | | | | | |
| --- | --- | --- | --- | --- | --- | --- | --- |
|  | **Chi square test** | **DF** | **P** | **RMSEA** | **(90% CI)^1^** | **TLI** | **CF** |
| **CFA** | 1187.2 | 1071 | 0.007 | 0.027 | (0.015–0.036) | 0.962 | 0.958 |
| **MG-CFA:** |  |  |  |  |  |  |  |
| **Sex** | 2304.3 | 2181 | 0.033 | 0.027 | (0.009–0.038) | 0.953 | 0.957 |
| **Number of years since graduation (months)** | 2310.9 | 2181 | 0.026 | 0.028 | (0.011–0.039) | 0.95 | 0.953 |
| **Two speciality subgroups^2^** | 2298.34 | 2181 | 0.04 | 0.027 | (0.007–0.038) | 0.961 | 0.964 |

DF: Degrees of freedom; RMSEA: root mean square error of approximation; CI: confidence interval; TLI: Tucker-Lewis Index; CFI: Close Fit Index; CFA: confirmatory factor analysis; MG-CFA multidimensional model confirmatory factor analysis.

^1^Corresponds to a 90% CI interval of RMSEA.

^2^Medicine and auxiliary; surgery and anaesthesiology

## **References**

1. Rosseel Y. lavaan: An R Package for Structural Equation Modeling. Journal of Statistical Software. 2012;48(2).

2. Holland PW, Wainer H. Differential item functioning.: Hillsdale, NJ, US: Lawrence Erlbaum Associates, Inc; 1993.

3. Svetina D, Rutkowski, L., & Rutkowski, D. . Multiple-Group Invariance with Categorical Outcomes Using Updated Guidelines: An Illustration Using M plus and the lavaan/semTools Packages. Structural Equation Modeling: A Multidisciplinary Journal, . 2020;27(1): 111–30.

4. Hu L, Bentler P. Cutoff criteria for fit indexes in covariance structure analysis: conventional criteria versus new alternatives. Struct Equ Modeling. 1999;6(1):1–55. .

5. Kenny D, Kaniskan B, McCoach D. The Performance of RMSEA in Models With Small Degrees of Freedom. Sociol Methods Res 2015(44(3)):486–507.

6. Boor K, Van Der Vleuten C, Teunissen P, Scherpbier A, Scheele F. Development and analysis of D-RECT, an instrument measuring residents' learning climate. Med Teach. 2011;33(10):820-7.
